# Infrastructure Revisited: An Ethnographic Case Study of how Health Information Infrastructure Shapes and Constrains Technological Innovation

**DOI:** 10.2196/16093

**Published:** 2019-12-19

**Authors:** Trisha Greenhalgh, Joseph Wherton, Sara Shaw, Chrysanthi Papoutsi, Shanti Vijayaraghavan, Rob Stones

**Affiliations:** 1 University of Oxford Oxford United Kingdom; 2 Barts Health NHS Trust London United Kingdom; 3 NICM Health Research Institute Western Sydney University Sydney Australia

**Keywords:** information infrastructure, structuration theory, video consultations, neo-institutional theory, organizational ethnography, hidden work, actor-network theory

## Abstract

**Background:**

Star defined infrastructure as something other things “run on”; it consists mainly of “boring things.” Building on her classic 1999 paper, and acknowledging contemporary developments in technologies, services, and systems, we developed a new theorization of health information infrastructure with five defining characteristics: (1) a material scaffolding, backgrounded when working and foregrounded upon breakdown; (2) embedded, relational, and emergent; (3) collectively learned, known, and practiced (through technologically-supported cooperative work and organizational routines); (4) patchworked (incrementally built and fixed) and path-dependent (influenced by technical and socio-cultural legacies); and (5) institutionally supported and sustained (eg, embodying standards negotiated and overseen by regulatory and professional bodies).

**Objective:**

Our theoretical objective was, in a health care context, to explore what information infrastructure is and how it shapes, supports, and constrains technological innovation. Our empirical objective was to examine the challenges of implementing and scaling up video consultation services.

**Methods:**

In this naturalistic case study, we collected a total of 450 hours of ethnographic observations, over 100 interviews, and about 100 local and national documents over 54 months. Sensitized by the characteristics of infrastructure, we sought examples of infrastructural challenges that had slowed implementation and scale-up. We arranged data thematically to gain familiarity before undertaking an analysis informed by strong structuration, neo-institutional, and social practice theories, together with elements taken from the actor-network theory.

**Results:**

We documented scale-up challenges at three different sites in our original case study, all of which relate to “boring things”: the selection of a platform to support video-mediated consultations, the replacement of desktop computers with virtual desktop infrastructure profiles, and problems with call quality. In a fourth subcase, configuration issues with licensed video-conferencing software limited the spread of the innovation to another UK site. In all four subcases, several features of infrastructure were evident, including: (1) intricacy and lack of dependability of the installed base; (2) interdependencies of technologies, processes, and routines, such that a fix for one problem generated problems elsewhere in the system; (3) the inertia of established routines; (4) the constraining (and, occasionally, enabling) effect of legacy systems; and (5) delays and conflicts relating to clinical quality and safety standards.

**Conclusions:**

Innovators and change agents who wish to introduce new technologies in health services and systems should: (1) attend to materiality (eg, expect bugs and breakdowns, and prioritize basic dependability over advanced functionality); (2) take a systemic and relational view of technologies (versus as an isolated tool or function); (3) remember that technology-supported work is cooperative and embedded in organizational routines, which are further embedded in other routines; (4) innovate incrementally, taking account of technological and socio-cultural legacies; (5) consider standards but also where these standards come from and what priorities and interests they represent; and (6) seek to create leeway for these standards to be adapted to different local conditions.

## Introduction

### Background

It has been 20 years since Star defined infrastructure as something other things “run on” and proposed nine defining characteristics: embeddedness, transparency, reach or scope, learned as part of membership, links with conventions of practice, embodiment of standards, built on an installed base, becomes visible on breakdown and fixed in modular increments [1,2]. The information infrastructure for health care needs to satisfy multiple use cases, such as personal health management, health care delivery (including assuring the quality and safety of care, audits, and billing), public health, research [3], and the formation of the scaffolding for a learning health system [4]. Sittig et al observed that this infrastructure consists not only of hardware and software but also the language of clinical applications, a human-computer interface, people who interact with it (including developers, support staff, staff-users, and patient-users), internal organizational features (eg, environment, policies, procedures, and culture), external rules and regulations, and the measures and metrics with which it is monitored [5].

In this paper, we seek to synthesize and extend these earlier theorizations. We begin by describing the clinical, organizational, and technical features of health care infrastructure. We then outline some central concepts in critical social science that inspired Star’s original work on infrastructure before offering a new theorization of health information infrastructure consisting of five key features. We illustrate this with a detailed contemporary case study of how this infrastructure influenced attempts to implement and scale up a video consultation service. After discussing these findings, we offer suggestions for those who seek to improve health services by introducing infrastructural innovations.

### An Overview of Health Information Infrastructure

Contemporary health care environments are saturated with technologies, many of which are highly sophisticated. However, health care infrastructure has a reputation for being convoluted, conservative, failure-prone, and lacking integration, for numerous interacting reasons [6-8]. Health care is complex, patients are unique, and the pace of change is rapid; new devices, procedures, service models, and policies are continually emerging, rendering elements of existing infrastructure obsolete. Safety (including the maxim “first, do no harm”) is an overriding concern, reflected in stringent design requirements and regulatory standards. A high proportion of clinical encounters are exceptions (ie, they deviate from the base case); technologies that assume an uncomplicated patient with a single, textbook condition and over-specify tasks and processes tend to be too brittle to support clinical work. Integration between specialties and sectors is expected but usually proves elusive, partly because it tends to be framed in technical and logistical (rather than socio-political) terms.

Health information infrastructure typically has hybrid roots, with incremental additions funded variously by local (public or private) providers, government, commercial suppliers, publicly funded research, and philanthropic sources [3,6]. It requires an exceptionally high level of security, reliability, and interoperability, and the resources to support repairs and developments to it (eg, redesigning routines and appointing and retraining staff). For all these reasons, the value proposition for heavy/traditional information technology (IT) (core, slow-moving, remote from a customer or patient [9]) in health care may be adverse, and the infrastructure supporting health care systems may be weak and fragmented. Some commercial suppliers prefer to focus on the more agile and less risky, customer-facing light/digital IT (including the growing wellness and wearables market).

In the United Kingdom, an advanced information infrastructure has emerged to support the public sector, nonprofit National Health Service (NHS) through in-house development, and a variety of contractual arrangements with different commercial suppliers [10]. Recent technological advances have made new service models possible, such as a commercially driven “doctor-in-your-pocket” video consultation service accessible via a smartphone app (which, controversially, bypasses much of the traditional NHS infrastructure) [11].

### The Critical Social Science of Information Infrastructure

Star was a cofounder of the “Society for People Interested in the Study of Boring Things” [2]. By this, she meant lists, classification schemes, ordering devices (eg, timetables), and the wires and connections that make an information system work. Many of these “boring things” inscribe the values, expectations, conflicts, and power relations of particular groups in society [2,12]. Developing standards, for example, is a social and political (ie, value-laden and power-charged) process in which a more-or-less agreed-upon version of the world is constructed and negotiated [13,14].

Star proposed that ethnographers can learn a lot by exploring how “boring” infrastructure plays out in real-world settings [1,2]. A primary focus of such work is the ever-present tension between the general (standardized) and the local (situated), which people attempt to bridge through articulation or tinkering (the steps taken to get things done as work unfolds in real-time, despite material limitations, regulatory constraints, imperfect data, conflicting priorities, reluctant colleagues, etc) [15], and via boundary objects (things that are differently interpreted and used by different groups, while retaining a shared sense of what the object is) [16].

As Berg and Timmermans have pointed out, the universal aspects of information infrastructure are not preexisting [17]. Instead, they need to be codeveloped through dialogue among those who will use, or be affected by, the infrastructure [17]:

…disorder preexists and precedes the emergence of order. The phoenix of universality rises from the ashes of local chaos. In the structure of these accounts, the local is the generic, natural state that is subsequently transformed; it is the unquestioned base from which the analysis starts.

Not only is order more arbitrary than is often assumed, suggest Berg and Timmermans, but any creation of order in one part of the system will create disorder somewhere else [17]. Because of this, the scaling up of a technology-supported service model to new settings is likely to be an unexpectedly tricky process and one that will not be easy to research, since ethnographic methods cannot be easily applied across multiple settings [18,19]. In sum, the health information infrastructure in many countries is characterized by state of the art individual technologies which need to interface with (but have often been designed with little awareness of) a legacy infrastructure and restrictive regulatory standards, all in the context of a complex, fast-changing, unpredictable, and underfunded service environment.

### A New Theorization of Health Information Infrastructure

#### Overview

In this section, we outline our theorization of health information infrastructure, which adapts and extends original work by Star [1,2] and subsequent scholars [3,5,6]. It consists of five essential characteristics, listed below.

#### A Material Scaffolding, Backgrounded When Working and Foregrounded Upon Breakdown

What Star called the installed base of infrastructure comprises both hardware and software as well as the rooms, desks, tunnels, pipes, and other things that host it. It is typically large and extensive (eg, allowing communication at a distance), but tends to drift into the background as it provides the mundane means to the ends of an organization, and because significant parts of it are buried, kept in a back office, or found in the cloud. To the familiar user, infrastructure is what Star called transparent (in the computer science sense of being invisible, taken for granted, and ready to hand). It does not have to be reinvented each time, but it becomes visible on breakdown (such as when a server crashes or a software upgrade reveals a bug) [2]. Breakdown in this context can also mean situations where the material or nonmaterial features of infrastructure prove too inflexible to allow a clinician to exercise autonomy to deliver care.

#### Embedded, Relational, and Emergent

Star observed that infrastructure is “sunk into, and inside of, other structures, social arrangements and technologies” [2]. She considered infrastructure a fundamentally relational concept, becoming real in relation to organized practices. Organizational knowing (the knowledge on which members draw when deciding how to act) is, to a large extent, tacit and embodied [20]. It draws on shared schemas and practices but also the material scaffolding of buildings, rooms, computers, records, charts, and other aspects of infrastructure [21]. The medical record, for example, is best conceptualized not as an isolated and static container of data but as a dynamic, evolving map of what is, was, and will be happening to the patient; this record links to other human and technological actors and to documents and practices [7]. This relational network evolves dynamically over time.

#### Collectively Learned, Known, and Practiced

Organizational work is complicated. It involves multiple actors who must share an understanding of context (primarily which technical and sociocultural actions are possible, allowable, and required) [22]. Such knowledge is built through collective learning and sensemaking, and it becomes embedded through both hard (eg, protocols or algorithms) and soft (eg, organizational routines, processes, practices, and norms) mechanisms. Indeed, organizational work can be conceptualized as multiple collective routines that are embedded within, and influenced by, one another; they are supported by shared material artifacts and social expectations that either harmonize or create conflict [23].

Maniatopoulos et al, drawing on Bourdieu and Schatzki, used the term “field of practice” to describe the nexus of interconnected and interdependent practices and arrangements, distributed across social, cultural, and material orders, that form the context for organizational work in a health care setting [24]. Infrastructure, according to Star, embodies such fields of practice and is learned as a part of membership [2]. The relationship between the physical (material) and knowledge/practice (nonmaterial) features of infrastructure is interdependent and reciprocal, as each shapes and is shaped by the other [24,25]. Both aspects of infrastructure, in turn, need to be differentiated from the purposive content of the interactions and practices they enable. A video consultation, for example, relies on infrastructure to provide its conditions of possibility, but the consultation itself also has its *sui generis* content intrinsic to its purpose. The conditions of possibility that the infrastructure provides will be more or less adequate to the clinician’s view of this purpose.

#### Patchworked and Path-Dependent

Hanseth and Monteiro described infrastructure as “a layered patchwork of components and associated routines which emerge historically” [26]. It is rarely installed or replaced wholescale, and it cannot be fixed all at once or globally. New components are continually added, and they must be designed for backward compatibility with existing components. Importantly, these legacy components are not just technical. They contain embedded standards and assumptions that reflect historical socio-cultural influences (eg, a drug ordering system that fails to consider nurse prescribing because it was designed when only doctors could prescribe). Legacy systems also reflect historical strategic decisions (eg, to invest, or not, in bandwidth, or to award an exclusive contract to a particular supplier), which set an organization on a particular path from which it is difficult to deviate. Because of such challenges, and because nobody is really in charge of infrastructure, changes to it typically take time and negotiation; they may involve substantial power struggles and unanticipated financial costs, but they may sometimes turn out to be impossible.

#### Institutionally Supported and Sustained

Institutional theorists distinguish between technical environments, in which organizations produce a product or service with a focus on effective and efficient performance, and institutional environments, which are characterized by the need to conform to particular rules and requirements to receive legitimacy and support [27,28]. As March and Olsen put it, “institutions reflect the routine way in which people do what they are supposed to do” [29]*.* In institutional environments, the technical components of the infrastructure are constructed and connect in a highly standardized fashion. Asking questions about which standards are used and where they came from can surface a narrative about the roles of, and conflicts among, societal macroactors, including government, industry, professional bodies, and others [2].

Scott identifies three institutional pillars that operate with health care: regulative (statutory and legal requirements/what we must do), normative (professional norms, values, and definitions of excellence/what we should do), and cultural-cognitive (internalized scripts/what we unconsciously recognize as what everyone does) [28]. The standards, norms, and scripts of a particular health care system (eg, what is deemed safe, medical-grade, evidence-based, high-quality, confidential, etc) are enduring and hard to change, more so because they are materially inscribed in infrastructure.

### Aims, Objectives, and Research Questions

Against this background, we sought to study health information infrastructure in a contemporary case study of innovation and change. Our theoretical objective was to explore, in the context of a heavily institutionalized health care environment, what infrastructure is, and how it influences technological innovation. Our empirical objective was, in partnership with front-line clinical and informatics staff at a self-proclaimed digital innovator hospital trust, to explore the challenges of scaling up a video consultation service across different clinical specialties and spreading this model to other sites. Our research question was: What was the nature of the infrastructure in this setting, and how did it shape (and become shaped by) the implementation of video consultations in different clinical and geographical settings?

## Methods

### Study Design and Setting

This study is a naturalistic case study with an action research component in Petroc Health (pseudonym), a UK hospital trust, along with three other trusts (anonymized as Eastern, Southern, and Northern), all of which are seeking to emulate the described model. Data sources (summarized in Table 1) included ethnographic field notes, interviews, documents, and material artifacts.

### Provenance, Management, and Governance of the Research

This work incorporated and extended the VOCAL (Virtual Online Consultations—Advantages and Limitations) study, which ran from 2015 to 2017. Its methods [30] and findings [31,32] (including details of ethics approval) have been published previously. An extension study called “Scaling Up VOCAL” began in 2017 with ethics approval from London Riverside Research Ethics Committee (reference 19/LO/0550; IRAS project ID 258679) and will complete in 2020.

In the early stages of this research, action research meetings were held approximately every three months. However, as the study progressed, these were incorporated into the trust’s existing governance structures, including a Transforming Services Together (TST) Outpatient Working Group, whose goal was to drive improvements in outpatient care. In 2018, a subcommittee of this group formed a Virtual Consultation Unit to focus specifically on local scale-up and a broader spread of video consultations. We supported these groups to articulate a theory of change, collect and periodically review data on the project’s progress, and amend ongoing plans and targets. The work included developing resources and horizon-scanning wider system issues and developments (eg, technology platforms, national policies). The research was overseen by a steering group with a lay chair and patient representation, and a separate patient advisory group gave periodic feedback.

The constraints of infrastructure at the microlevel of the clinical consultation have been described in previous papers [10,32]. For this paper, we focused primarily on efforts to achieve translation by organizational actors at the meso level, that is, to engage interest and mobilize other actors (human and technological) to achieve the task of making video consultations business as usual [33].

### The Case

The leading case site, Petroc Health, is a large, multi-site acute hospital trust and a recognized digital innovator, located in a deprived and multi-ethnic part of London. At the time of this study, NHS organizations were experiencing year-on-year reductions in their budget at a time of rising need, worsening staff shortages, and political and economic uncertainty. All departments were under pressure to deliver services more cost-effectively, and technologies were depicted, both nationally and locally, to achieve this. Outpatient clinics at Petroc were crowded, and there was huge pressure on the availability of consulting rooms. Travel between the trust’s dispersed sites was difficult and time-consuming. The did not attend rate for some outpatient clinics exceeded 50%. Video consultations were introduced initially to try to reduce did not attend rates, though only some clinicians engaged with the new model, and many patients were considered unsuitable for the video option for clinical (eg, high-risk) or socio-cultural (eg, low health literacy) reasons [31,32]. A cyberattack in 2017 led to a devastating and widely reported collapse of the trust’s information system [34].

Eastern, Southern, and Northern NHS trusts, based in different parts of England, had all approached Petroc intending to emulate their virtual consultation service model in one or more clinics. As part of the Scaling Up VOCAL project, members of the Petroc team supported clinicians and managers in these trusts, which presented different cases in terms of size, geography, patient population, and use of digital technology.

### Participants, Sampling, and Dataset

We followed the introduction of video consultations initially in three clinical departments (diabetes, antenatal diabetes, and cancer surgery) and subsequent attempts to roll this model out to an additional ten departments in Petroc Health, including hematology, endocrinology, rheumatology, and neurology, and to a further six departments in the other three sites. Data were collected at a macro level (eg, national stakeholder interviews, policy documents), meso level (interviews, field notes, and internal documents from organizational ethnography), and microlevel (eg, observation and video-recording of clinical consultations). The full dataset and its contribution to our analysis and theorization are shown in Table 1.

**Table 1:** Overview of multi-level data collection and analysis (including the earlier VOCAL study).

Data level	VOCAL^a^ study (2015-17)	Scaling Up VOCAL study (2017-20)	First-order interpretation	Higher-order categories
Microlevel study of virtual consultations and efforts to deliver these on a clinic-by-clinic basis	30 videotaped remote consultations 16 face-to-face consultations (field notes linked to those)	Analysis of design and material properties of five video technology platforms used across participating sites: Adobe Connect, Skype (consumer), Skype for Business, Attend Anywhere, Microsoft TEAMS. Interviews and think aloud observations using the technology within six clinical services (diabetes, endocrinology, hematology, rheumatology, orthopedics, cancer).	What is said and done in consultations, and the local setting-up of video consultations How technology influences clinical work and how individual agency influences technology use, including examples of paradoxes (eg, a small change in technology has a significant effect), breakdown (where infrastructure becomes visible). invisible work, and articulation (eg, tinkering to deliver a service despite local contingencies)	Institutional assumptions built into the material and technological infrastructure (eg, about the capability of users, access rights, costs and payments, privacy and consent laws, and nature of clinical work) Internal social structures (habitus) of clinicians, such as personal and professional codes, and perspectives on illness. Specific knowledge of particular patients, and local system knowledge How the tension between standardization and contingency plays out as clinicians use technologies in clinical care (or find they cannot use them as anticipated)
Mesolevel study of organizational change	24 staff interviews 300 hours of clinic observation 16 trust-level documents. Throughput and demographic data (eg, number and percent of consultations done via video).	Main site: 150 hours of ethnographic observation, 23 interviews with 17 staff, activity, and patient demographic data for six participating clinics. Secondary sites: 20 hours of ethnographic observation, 14 interviews with ten staff	Departmental-level case studies of efforts to introduce and mainstream a video consultation service Human actors’ attempts at translation (problematization, interessement, enrolment and mobilization [33]) How competing interests and agendas played out in each case study	How organizational values, traditions, and routines (embodied in scripts) change over time, and why they endure Role of individual agency in both embodying and challenging institutional structures How the micropolitics of the institutional setting shapes and constrains an implementation effort
Macrolevel study of the wider context for introducing video consulting	48 stakeholder interviews 50 national-level documents from 2000-2017 (including policies, guidance, and national-level announcements)	One further stakeholder interview Ten additional policy and guidance documents published 2017-19	Historical and policy drivers for, and barriers to, the introduction of video consultations in UK’s National Health Service Reasons for emergence of alternative service models involving video consultations (eg, in new models of general practice)	Institutional pillars which help sustain traditional face-to-face modes of consulting, including regulative (laws, tariffs, standards), normative (eg, professional, ethical codes and definitions of excellence), and cultural-cognitive (master-narratives of what a medical consultation is and how to behave in it) How these institutional pillars are inscribed in the National Health Service information infrastructure

^a^VOCAL: Virtual Online Consultations—Advantages and Limitations.

### Theoretical Framework

As described previously in our VOCAL publications [30-32], and in common with Orlikowski [35] and Scott [28] (but in contrast to actor-network theorists who prefer a flat ontology [33]), we used a theoretical approach which assumes both microactors (human and technological agents) and macroactors (within broader social structures) who interact and coevolve in a dynamic and recursive (mutually-influencing) way [36].

Stones’ adaptation of Giddens’ work in strong structuration theory [37], and its extension by Greenhalgh and Stones to include technologies [38] considers both external social structures (such things as laws and regulations, societal and professional norms, and socio-cultural expectations) and how those structures are internalized as habitus by people (as knowledge, acquired dispositions, experience, normative orientations and morals, and patterns of learned behavior) and by technologies (as encoded rules, standards, access controls, and role assumptions). Teasing out these internalized social structures helps us to theorize the different priorities and agendas of different individuals, depending (for example) on their professional allegiance, role and status in the organization, prior training, experience, and so on.

In deciding how to act in any social situation, human actors – variously positioned within the configurations and hierarchies of organizations – draw on both their habitus and on their assessment of the here-and-now strategic terrain. There will often be a tension between the generalized socio-cultural frames of habitus and knowledge of the immediate field of practice. Some actions will appear possible, expected, and required, while some will seem impossible, inappropriate, or unimaginable. Similarly, the internalized social structures of technologies will (in some cases) extend what can be contemplated and achieved, and (in other cases) place limits on what is possible.

Like actor-network theory, strong structuration theory envisages humans and technologies as linked in dynamically evolving networks, which are inherently unstable but can potentially become stabilized when a particular configuration of people and technologies becomes aligned. The institutional aspects of health information infrastructures described above confer a high degree of stability on them, meaning that introducing a significant change to infrastructure requires substantial realignment of people, technologies, standards, procedures, training, incentives, and others. This process occurs in four stages: problematization (defining a problem for which a particular technology is a solution), interessement (getting others to accept this problem-solution), enrolment (defining key roles and practices in the network), and mobilization (engaging others in fulfilling the roles, undertaking the practices and linking with others) [33].

### Analytic Approach

We took a two-stage approach to data analysis. First, we analyzed interviews, documents, and field notes thematically to gain familiarity and identify broad themes and categories. Second, we used reflection and team discussion to undertake a more theoretical analysis using technology-enhanced strong structuration theory as a guiding framework. We sought to identify macrostructures (Scott’s regulative, normative and cultural-cognitive institutional pillars [28]), how these structures were internalized within the habitus of human actors (as beliefs, values, attitudes) and in technological actors (as algorithms, access privileges, menus, quality of particular functions), and how they shaped and constrained action in particular encounters and events. We also considered how human actors sought, successfully or unsuccessfully, to change and stabilize the sociotechnical network in their effort to routinize and scale-up video consultations.

To hone our focus on health information infrastructure, we sought to construct rich ethnographic accounts of cases within the case (such as recurring problems with bandwidth) that illustrated and explored one or more of the five characteristics of infrastructure set out above. We followed some guiding principles of the ethnography of infrastructure suggested by Star: surface master-narratives (the overarching discourses that shape decisions), apply infrastructural inversion (eg, foreground things that are usually kept in the background), surface invisible work (eg, work done by low-grade staff such as secretaries and administrators), and study paradoxes (eg, why a simple change makes the whole system unworkable, perhaps because it generates additional hidden work) [39]. We also built on Star’s original approach to add a more specific institutional dimension to the analysis, considering, for example, how institutional roles and identities generated particular agendas and priorities and showing how these agendas and priorities sometimes clashed.

We used narrative methods to organize our different data elements into a coherent temporal account, which made sense of unfolding actions and events in both a local and broader context [40,41]. Narrativizing helped illuminate the unique and subtle socio-technical interdependencies hidden within technology-supported change programs [42] since the significance of seemingly mundane or unrelated data items (eg, a short exchange of emails, field notes from different clinics, minutes of meetings held over months or years) often became evident only when incorporated into a story that spanned both time and space. This is especially true since infrastructural problems typically took months or years, not days or weeks, to resolve (and in many cases remained unresolved). The narrative form also allowed us to tease out and emplot micropolitical events and actions (such as how both conflicts and collaborations emerged between clinical services and the IT department).

## Results

### Description of Dataset and Overview of Findings

Our final dataset (to date) of 450 hours of ethnographic observation, 61 staff interviews, over 50 wider stakeholder interviews, and a large body of documents (Table 1) was characterized by a striking prominence of “boring things” [1,2], such as small material details, protocols, standard operating procedures, and seemingly banal rules and regulations. Repeatedly, implementation and spread efforts were stalled or distorted by things that were so mundane we initially hardly noticed them. In the early months of our research, we unconsciously backgrounded these “boring things” as our research gaze was drawn to more conventional subjects of ethnographic observation: the talk and action of human actors. However, as the study unfolded, we recognized a recurring pattern: human actors often found themselves unable to act in a way that would have helped implement and spread the video consultation model. It was when we began to explore the causes of these delays and bottlenecks that the infrastructural themes described in this paper began to emerge.

Below, we describe four examples of how infrastructural issues repeatedly stalled this translational effort within Petroc Health and the other provider organizations that were seeking to emulate its video consultation model.

### Selection of a Platform to Support Video-Mediated Consultations

This subcase describes various challenges with four different platforms that have been considered over the years for supporting video consultations in Petroc Health. It illustrates many of the features of infrastructure described in the introduction, including the importance of its material features and dependability, its embeddedness in systems and organized practices, the incremental, patchworked, and path-dependent way it is built and added to, the close interdependency of technical components and standards (eg, the need for top management endorsement that its components are approved, safe, and formally supported by the information and communication technologies [ICT] department), and the micropolitical nature of software choices (especially how senior management is minded to reject what clinicians see as a more patient-centered platform because it is less compliant with regulatory standards).

Video consultations were first piloted in the diabetes clinic in 2011 by a consultant diabetologist, anonymized as TW. A commercial videoconferencing tool, Adobe Connect (Adobe, San Jose, California, United States), was initially used because the project funder (NHS Choices, part of the English Department of Health) had already obtained these licenses. Like most corporate videoconferencing tools, Adobe Connect required a host to set up a conference call slot using a calendar schedule. It also offered session recording, instant messaging, and screen sharing (intended for presentations). The trust’s ICT department were reassured by the program’s end-to-end encryption and the company’s security credentials. Clinicians chose not to use the video-recording function, and the consultations were not captured on the patient’s electronic record except via conventional clinical notes. Screen-sharing was occasionally used to show patients on-screen data, such as test results.

Adobe Connect’s video connection was technically reliable but fraught with difficulties. For example, the need to invite patients to a conference meeting by sending a URL link added a layer of administrative overhead (hidden work) for the clinician. The software was unfamiliar to both clinician and patient, and it used a metaphor (conference room) that jarred with the institutional purpose of the call (a confidential medical consultation). While the new video consultation service was popular with patients and staff, the platform was not.

Accordingly, the diabetes team tried using the consumer version of Skype (Skype Communications S.A.R.L., Luxembourg City, Luxembourg) as part of a one-year feasibility study funded by the Health Foundation, a large medical charity. This decision proved to be more popular. Many patients already used Skype, and the rest generally knew someone who could show them how to download and use the platform. In 2013, the team obtained further funding from the same organization to explore the use of Skype-based consultations in routine care. Unlike Adobe Connect, Skype is an open-ended, point-to-point, video call application that allows users to customize the structure of the interaction. In addition to booking video consultations, it allowed patients to see when the clinician was online and send them an instant message. This was a function that was seen as empowering patients who had been reluctant to attend clinic:

The advantage of seeing who is around is useful for us – it has allowed a lot of ad hoc contacts with patients who never turn up physically to clinics, but Skype the nurse to say “Hi – saw you were online and wanted to check this with you.” This has contributed significantly to patient engagement with the service and them taking control.

TW

By 2015, Skype consultations had become business as usual in TW’s diabetes clinic. However, our ethnographic observations showed that this apparent embedding was the result of elaborate workarounds by clinical and administrative staff for aligning the clinical consultation with documenting attendance and booking a repeat appointment. In a regular consultation, this would have been triggered by the patient physically appearing at the booking desk. This finding illustrated the deep embeddedness of the clinical routine within other, more administrative routines [23].

The Skype demonstration project described above had been informally supported by the ICT department (who had installed Skype on outpatient computers as a favor to TW, who was well-liked). The project was popular and widely highlighted (the UK Secretary of State for Health, for example, used it as an example of digital innovation in high-profile speeches [43]). However, efforts to roll out this model to other departments within Petroc Health quickly stalled when senior management raised concerns about the technical and regulatory implications of using the consumer version of Skype. They pointed out the lack of information governance rules or guidance on the use of Skype within the NHS, the absence of service level agreements or protocols for managing installation and upgrading of the software, and the unknown local impact of video streaming on network connectivity. The practical impact of the absence of agreed standards and procedures was that, for example, a clinician would arrive to do a booked video clinic and find that because of a forced upgrade overnight, Skype no longer worked on the local terminal. This reflected the fact that some aspects of the infrastructure were controlled by players outside the organization, such as Microsoft. This problem could be fixed only when the clinician could cajole someone from the ICT department into attending in person and manually overriding the block.

TW used her experience, skill, and personal qualities to work through Latour’s four stages of translation in her effort to transform and stabilize the socio-technical network needed to make video consultations a reality: problematization (defining the problem as accessibility of services and a particular technological intervention as the solution to this), interessement (getting others to accept this problem-solution, specifically by engaging top management and board members as well as her fellow clinicians), enrolment (defining key roles and practices in the network, such as establishing a cross-departmental working group that included ICT and business elements), and mobilization (engaging others in fulfilling the roles, maintaining dialogue, and creative troubleshooting).

As part of our action research, we helped produce the standardized procedures and documentation demanded (and subsequently approved) by senior management at Petroc Health. This, along with TW’s perseverance, led to Skype eventually being formally approved for running video consultations across the trust, which was a victory over the reluctant senior management. In 2017, its rollout formed part of the Transforming Services Together program. This strategic-level initiative was coincidentally given a boost because upgrades to some parts of the trust’s internet network were occurring as part of a separate initiative to support multidisciplinary team meetings between different sites using a different video-conferencing technology. This serendipitous event illustrates that infrastructure, as defined by Star, is a fundamentally relational concept, the reciprocal embeddedness of infrastructural elements, and the patchworked and path-dependent way in which the installed base evolves.

By the end of 2017, various additional videoconferencing applications had become available, some of which had been purpose-designed for medical use, and Skype was coming to be outdated and limited in comparison. Initially, there was little appetite by the project leads to replace Skype as the preferred platform, as they felt that after much effort, they had secured the necessary changes for supporting this particular software (the standard operating procedures and information governance approvals, for example, were specific to Skype).

However, during 2018, the trust encountered several technical problems running Skype (described further in subcases 2 and 3) and began to consider moving to a different platform. There was much discussion about the possibility of switching to the licensed version of Skype, Skype for Business, which at the time was the package of choice recommended by NHS Digital. Eventually, however, a trust-level decision was made to pilot Microsoft TEAMS (Microsoft, Redmond, Washington, United States) (a workplace collaboration tool with video call functionality), on the basis that this would form part of the Microsoft Office 365 package, which the ICT department was already planning to purchase on behalf of the trust (another example of the emergent, patchworked, and path-dependent nature of the installed base). Furthermore, our stakeholder interviews with Microsoft executives had revealed a plan to adapt the TEAMS software for clinical settings, including video consultations, and their plans to integrate this function with other parts of the electronic record. From May 2018, a series of meetings occurred between Petroc Health’s top management and Microsoft until a third-party broker organization was contracted to negotiate and mediate formal agreements.

During the same period, the project team became aware of another technology platform, Attend Anywhere (Attend Anywhere, Melbourne, Australia), which was purpose-built for clinical use. It had a design focus on emulating clinic workflows (eg, a single button on the trust website enabled patients to gain access to a virtual waiting room, potentially managed by a receptionist, for the patient to be joined virtually by the clinician when ready). Attend Anywhere had been developed by an Australian SME and was already being implemented in several sites in Scotland. In May 2018, Attend Anywhere was procured centrally by NHS Improvement to pilot within selected NHS trusts in England.

Petroc Health volunteered to be one of these pilot sites, creating an opportunity to explore and evaluate both Attend Anywhere and Microsoft TEAMS in parallel, and work out the business case for these two very different options (one custom-designed for this purpose but requiring purchase of additional licenses after the pilot period; the other a product that was free as part of a wider package but would need extensive customizing). At the time of writing, the Attend Anywhere versus TEAMS pilot is ongoing.

### The Introduction of Virtual Desktop Infrastructure Technology

Our next subcase describes how a technology (virtual desktop infrastructure [VDI]) introduced with the goal of improving efficiency and security through a standardized, lean (thin client), and centrally-controlled process led to both anticipated consequences (greater central oversight and control of local activity, some efficiency improvements for the IT department) and unanticipated ones (local breakdowns and knock-on problems elsewhere in the system). For poorly understood reasons (a hoped-for panopticon view never materialized), the Skype video consultation platform became unreliable and lost safety-critical audit-trail data, such as text messages and missed calls. At one point, the video consultation service could only be sustained through a single legacy terminal in one room that entirely bypassed the modern VDI solution. As Star predicted, this paradox proved revealing.

During 2016, the rollout of virtual clinics using Skype as part of the trust’s Transforming Services Together program, helped by a strongly positive national policy context, was primarily driven by TW, who had introduced the model in her clinic back in 2011. As part of this initiative, Skype had been made accessible on clinic computers for approved services that had been brought into the rollout initiative. Because of pushback from the overstretched IT department (who required more resources and wanted clear governance processes established before agreeing to a full scale-up), it was otherwise not supported on trust computers. By mid-2017, ten video consultation services were approved across Petroc’s five hospital sites.

At around the same time, many of the hospital computers (referred to by the ICT department as fat clients) in Petroc’s main central London site were being replaced with VDI units (thin clients). VDI is a virtualization technology that hosts a desktop operating system on a centralized server; the user’s VDI profile can be customized and run on any machine within the organization. The VDI deployment was a significant undertaking, with plans to cover all five hospital sites over three years. It was seen by ICT leads as a way of improving network efficiency, security, and cost-effectiveness, as well as providing clinical staff with a more consistent computer experience when working across different sites:

From an IT side of things, it is easier to manage because you don’t have to update the machine every time there is an update to Windows, or an application. It’s all done centrally. So, from an IT perspective it is easier to update and proceed. Also, for security. I mean when we got the cyberattack [May 2017], we got affected really badly. And so, all the security is managed on the server side, so it can’t get infected on the individual machine. So, there’s a lot of factors in there.

ICT Service Delivery Manager

In this new VDI environment, Skype needed to be installed centrally on individual staff members’ user profiles, as opposed to manually on specific computers. The clinical teams affected were assured that Skype would still be accessible through their personal VDI, and arrangements were put in place to install the application onto relevant staff VDI profiles.

By September 2017, VDI had been deployed across two hospital sites covering 2000 users. Only one non-VDI PC remained within the Diabetes center where TW was based. When she first logged onto her VDI profile, she was relieved to find Skype still available on her desktop. For two months, she continued to run video appointments as part of her weekly clinic, which constituted 10-20% of her follow-up appointment activity. However, in November 2017, she started to experience a problem where her Skype contact list would completely disappear. It was as if her Skype account had been completely wiped, which made it impossible to run video consultations, as both parties need to be established contacts. The history of all Skype interactions with that patient (including text messaging, contact requests, and missed calls) also disappeared.

As Skype appeared to work normally on the one remaining standalone PC, TW attributed the problem to VDI deployment. She emailed the ICT helpdesk seeking urgent assistance, anxious to retrieve her contact list before her next set of appointments. The ICT Service Desk Manager (after discussing with the Network team) concluded that Skype would not work on VDI and advised TW to continue running video appointments using the one remaining PC. However, this was not a realistic option, since TW did not have the authority to use a specific room:

Clinic rooms are not yours. You only use the one room allocated to you. Someone else might be using it.

TW

She escalated the issue, emailing the ICT manager (anonymized as GF) along with other senior members on the TST program, emphasizing the “serious clinical risk” of this problem and implication for their plans to scale up the use of virtual consultations across the trust. The ICT manager recognized the problem and assured TW that efforts were being made to get Skype working on the VDI.

Through December 2017 and January 2018, various members of the ICT team became involved in addressing the issue. As a temporary workaround, they set up a User Acceptance Testing (UAT) pool on TW’s VDI profile, from which she was asked to run Skype. The UAT pool is effectively a separate virtual machine used to run tests and reconfigure settings without impacting the wider live production VDI environment. Such pools had also been developed for other applications that were either new or posed problems for the VDI environment.

This period was a “constant irritation” for TW. Determined to keep her video clinic running, she continued to have video appointments with selected patients, while engineers sought to test and troubleshoot different ways of synchronizing her local Skype application with her central account. At times they managed to retrieve the contact list. However, often, it would disappear when she rebooted the machine, requiring further assistance through the ICT helpdesk.

As time went on, TW made sure this remained a priority issue on the Transforming Services Together agenda, raising it at meetings and sending group emails to various ICT and strategic actors. The ICT team continued to investigate but could not provide a clear idea of how or when it would be resolved.

By February 2018, one of the End User Computing engineers (anonymized as OB) who had been tasked to lead on this issue began to make some progress. By shadowing TW and reviewing background operating processes while she used the Skype application through her VDI profile, he identified that while some of her contacts appeared on loading the application, many did not. After some time, he worked out that the local machine was saving a profile of any Skype contacts used. If the same user logged in again on that machine, those contacts would be found again, and this would prevent additional contacts from the user’s wider Skype profile being copied across from Microsoft Cloud. Having identified the problem, OB anticipated that a fix could be found.

In February 2018, OB and other members of the ICT team invited TW to their office to show her how to run the Skype application from the UAT profile they had installed. OB documented his fix in a Word document as a script for the service support team to use in future cases. While TW was able to return to using Skype more readily, she now had to relaunch Skype every time she wanted to use it (to sync it with her online account), and from a specific local drive on her UAT pool (where the fix was implemented), rather than letting it run in the background. She considered this clunky compromise worthwhile to ensure the contact list remained intact and up to date.

Unfortunately, this promising fix did not last long. After a few months, TW’s contact list disappeared again. TW contacted OB, who reimplemented his script, which rectified the issue. Nevertheless, after a few weeks, the problem occurred again. In the months that followed, it became a routine practice for TW to call upon various ICT engineers whom she got to know, asking them to reimplement OB’s script whenever the issue occurred. This was frustrating and awkward, not least because the Skype clinic ran from 8:45-9:30 am, but the ICT helpdesk did not open until 9:00 am. During that time, TW purposefully restricted the service to existing and familiar patients in the hope that a more permanent solution would be found.

However, in August 2018, this issue was further confounded by another problem. TW noticed that Skype was running particularly slowly, and it also slowed down the other clinical applications. Sometimes it ran so slowly that it was impossible to use. On other occasions the application crashed altogether:

The whole thing would slow down. And that was terrible because the patient would call in. And I could see they were calling in. But I couldn’t even accept the call because the whole thing was so slow. By the time I got the cursor on the icon and click, the patient would call off. So, it was just awful. They could see I was online. They could see that I would have seen the call. But I wasn’t picking up the call. And normally, when that happens – because if I am talking to another patient, I would type them a message to say I’m busy I will reply in ten minutes. But in this case, I wasn’t even able to do that. The whole thing was terrible. Even if I was able to manage to connect. Everything was slow. I couldn’t open patient records.

TW

Consequently, TW and other members of her team stopped running video appointments completely. TW and other members of the rollout program were beginning to consider other technology options (see subcase 1). However, this would take time to establish. In the meantime, TW was asked to launch Skype on her VDI profile every day for six weeks and keep logs of her experience to try and develop a clearer picture of what kept happening. Partly because the problem largely resolved over the Christmas period (when network usage dropped), the ICT team concluded that the Skype application used more memory than had been anticipated and increased the central processing unit (CPU) for TW’s UAT pool. While this appeared to resolve the problem, TW considered this fix too precarious to justify expanding the service to new patients or encouraging other staff to use it. The fact that she was not under pressure to push the pace of implementation illustrates that the project, while formally Trust policy, was not an overriding priority.

A member of the ICT department summed up the prevailing sense of confusion and impasse:

The challenge we have with VDI is lots of patchy fixes and never really clear what exactly they have done to fix it… And we have hundreds of applications that we use across the trust. And this will be happening with lots of other programmes. … It is very very difficult to isolate what is happening. A lot of it is feeling around in the dark as to what may or may not be happening. Even when you get an answer, it will change tomorrow…

ICT Project Manager

At the time of writing, the trust, and the IT support team are still struggling with the challenges of VDI technology. A reviewer of a previous draft of this paper suggested that the kind of technical analysis described in this case could have been done in a more automated way using situated analytics had the IT department been aware of such approaches [44], though these may well bring their own challenges.

This case illustrates how even in digital-exemplar health care organizations, breakdowns are frequent, and repairs are not merely technological but also social and political. The clinical entrepreneur (TW) achieves repairs by using her status and relationships within the organization to mobilize the ICT support infrastructure and persuade support staff to agree to bespoke solutions (eg, allowing the use of the legacy desktop machine to run Skype). In other cases, lower-status junior doctors and nursing staff had less power and influence in this regard, and in that sense, carried a heavier infrastructural burden (ie, their work was more vulnerable to constraints and delays when breakdowns occurred). Such staff were, for example, dependent on a response from the generic IT helpdesk support email address (because they either lacked the seniority to contact anyone else directly, or lacked knowledge of whom to speak to), and progress tended to be slow unless the problem could be escalated to someone with better connections.

### Video Call Quality in a Peripheral Hospital

This subcase relates to a rheumatology clinic in one of the peripheral hospitals of Petroc Health (anonymized as Eastern General). It illustrates the patchworked and incrementally fixed nature of health information infrastructure. Previously a separate, small district general hospital 20 miles away on the outskirts of London, Eastern General merged with the main Petroc Health Trust in 2013. As this case illustrates, the distant site has become swept up in the wider modernization effort of this digital innovator organization, a perhaps typical experience for the smaller and weaker partner in a merged organization. The learning curve for the team at Eastern General is long and steep. For the clinicians in the narrative, the goal is not merely to make the video connection work technically but to ensure that it works reliably and to a sufficient standard to support professional work. Despite many time-consuming test calls to try to optimize the technology, it fails on the day, and she, in turn, feels she has failed her patients.

Eastern General was widely viewed as both clinically and technologically backward by those in the main central London site. In September 2017, CB, the physiotherapist, met with GT (Service Delivery Manager) and LT (Patient Pathway Coordinator) in the staff meeting room to test Skype on her computer and familiarize herself with the application. Her first video appointment was booked for the following week. After spending some time tinkering (eg, finding USB ports that worked, positioning webcam, setting the correct audio output devices), navigating (eg, how to find contact, initiate calls, send messages) and test calling (using the Skype test call audio playback service), they got the technology to work.

After months of preparation, the team was excited to finally launch their new video clinic, which was the first on this site. For six months, GT had worked with the local Service Manager (FI) to get everything in place. Among other things, they had to submit requests to the ICT department to incorporate video appointment slots into CB’s electronic scheduling tool, generate new appointment letters, and gain access to the Skype application on the clinic desktop computer (the peripheral hospital was yet to be modernized with VDI technology).

On the morning of the first video clinic, CB arrived at work earlier than usual. She needed to collect the webcam and headset from the administrative office, load up Skype, and check it was working on the clinic computer. The one video appointment was deliberately scheduled to take place at the end of her clinic so as not disrupt her face-to-face appointments. When that time came, Skype was slow to respond. When the patient answered CB’s call, his face was severely pixelated. At times, the video froze or dropped altogether. The audio was also broken and distorted. Clinician and patient struggled on, but only by disabling the video function at both ends.

CB did not want a repeat of this challenging and embarrassing situation. She decided to run some more test calls before the following week’s clinic. She wanted to know if this was a one-off glitch or a problem with the hospital connection that was likely to be recurrent; she strongly suspected the latter. Over the next few days, she found time to perform test calls (with colleagues and members of the implementation team) from the consultation room, with variable and inconclusive results. She would have liked to have done more tests, but time was limited because the consultation room was rarely free to use, and she needed to fit in with the availability of the person on the other end. The following Tuesday, she ran another video appointment with a patient. This one was just as bad. She decided to discontinue video appointment bookings until she could trust the technology.

And I guess it’s frustrating because you are messing around for a few minutes and it doesn’t look very professional for the patient, where I’m having to hang up the call and pick up the call again, and say why can’t I see them.

CB

FG (Service Manager) escalated the problem with the ICT manager (GF), who passed it to the program lead responsible for supporting the roll-out of video clinics (BN). For the ICT team, this was not a simple technical fix. There appeared to be no objective way of assessing bandwidth requirements; they could only “suck it and see.” Video streaming was a new demand on Eastern General’s overstretched network, which had been designed historically for much lighter information traffic but had recently become (organizationally and technically) part of the digital innovator Petroc Health. Various attempts to modernize Eastern General by installing new technologies had not gone well:

Eastern General has a very – a network under pressure. It’s organically grown to support the service but not necessarily with the nuts and bolts required on the network. So what we have is a lot of people hanging off the network more than what it can manage. […] People are competing for the bandwidth that’s there […]. What you’ll get is limited bandwidth at different locations depending on the size of the fibres that have been put in, or the connections that have been put in. […] [T]hat bit of spaghetti may have been a 10 meg link. Whereas some people had, I don’t know, linguini which was the fatter one, and that’s a 100 meg link. And then you sit on a much bigger pipe which is the fibre. So if you sit there, you’ve got a weaker link.

ICT Support Manager

The above quote illustrates how the clinicians, patients, managers, and relevant ICT people, along with elements of the technical network, have all been enrolled into an effort to get a new, video-based rheumatology service up and running at Eastern General, but the known limits on capacity in this historically low-tech site were somehow not factored into the planning. Batteau [45] has pointed out:

Heterogeneity of expectations, artifacts, infrastructure, support, skills, management, and system planning … is an inevitable consequence of the variable rates of diffusion [of innovations] in a large-scale technological system within an articulated social field.

The task is to rise to the challenges that are posed by such inescapably complex relations and processes.

CB continued to try test calls when she could, to gain a better idea of how the video call quality varied. On one occasion, this was done with an ICT engineer present, who went on to speak with the server team to ascertain how call quality corresponded with wider network usage. Despite initial plans to run these tests more frequently, in practice, it was too difficult to organize and coordinate them alongside the availability of the clinic room.

With limited data and multiple interacting influences (eg, variations in network capacity and traffic within different sections, and at different times, across the network, and the CPU demand of Skype and other systems running on the local terminal), it was difficult for the ICT department to confidently establish the underlying cause of the problem. GF decided to postpone any further deployments of video consultation technology at Eastern General. The hospital was due to have two major IT upgrades within the year, so it made sense to wait before progressing with the video clinics. The ICT support manager was upbeat about this:

In the next six months they will have a whole new network and their Skype will be singing and dancing. Because effectively we are giving them a motorway in terms of provision of bandwidth.

For the ICT team, their experience at Eastern General highlighted the value of what they referred to as a “controlled rollout.” This involved working with a few services, each running a small number of consultations, to understand how Skype impacts the network and manage or restrict activity where necessary.

For CB and her team, it was felt that some momentum still needed to be maintained for their new virtual clinic service. CB believed video quality was far better during her Friday morning clinic than the Tuesday clinic, perhaps due to less demand on the network at this time. She asked GT to prepare and submit another clinic template change form to incorporate video slots into her Friday clinic.

By November 2017 (which was before the two major upgrades), the new (Friday) video slots had been implemented, and CB began offering video appointments. On 30 November, she ran two successful video appointments. The video quality, though not perfect, was enough to run the consultation. In the knowledge that the Eastern General network will be improved at some point, she has continued to press ahead cautiously with Skype in a small number of carefully selected patients.

CB’s frustration reflects several features of what we have previously (following MacIntyre) called medicine’s internal goods [46]: being available for the patient (as a professional standard, the risk of not being available must be zero), assuring continuity of care (rheumatology patients generally have lifelong conditions and stay with their specialist clinician for years), and providing professional presence (via a technically adequate video connection). Given that the unreliable connection jeopardized all these internal goods, it was small wonder that the clinician felt that “it doesn’t look very professional.”

### Access Rights to Use Video Conferencing Software

Our final subcase highlights how the embeddedness of infrastructure in local systems poses challenges to spreading a technology-supported service model to other organizations. Adoption required distributed reconfigurations across multiple levels of practice, which were reciprocal and interlocking. The technical infrastructure (including a licensed Skype technology acquired through a previous purchasing decision) afforded and constrained actions in ways that played out differently to the main case site, in which particular organizational routines, processes, knowledge, standards, and workarounds had emerged. In this last case, information governance concerns and standards constrained technological choices and slowed the implementation of solutions.

Southern Trust, a large, multi-site provider in a university city that had won awards as a digital innovator, was supported by the Video Consultation Unit at Petroc Health to try to introduce video consultations in various clinical services. One of these was a sarcoma (bone and soft tissue tumor) clinic, where a consultant surgeon, SB, led the implementation. After signing up, implementation was put on hold for several months due to a significant increase in clinic demand alongside senior staff changes and recruitment delays.

In April 2018, after SB was able to secure some additional operational staff time, progress was made to set up the technology. Skype for Business had been part of a recent NHS Mail integration, so it was available free to all staff users. This platform offered similar management and security functionality as Adobe Connect, but also interfaced with Skype and allowed people without an account to join as guests, avoiding the need to set up patient accounts. The additional call encryption capabilities and integration with NHS Mail (secure email accounts for NHS staff) were also considered an advantage over the consumer version of Skype, which had not been designed with the privacy needs of medical consultations in mind.

By September 2018, Skype for Business was installed on a dedicated laptop (specifically purchased for running video appointments), to be held by an administrator and taken to the clinic by SB. This was because most clinic computers used VDI profiles, and there were uncertainties about the reliability of Skype on these (reinforced by the experiences at Petroc). Use of the laptop required governance approvals with the recently introduced Data Protection Impact Assessment tool to comply with the UK’s General Data Protection Regulations.

SB identified some suitable patients to run his first video appointments but wanted to run dummy calls and familiarize himself with the technology before booking them in. However, his series of attempts to test the technology repeatedly failed. The application was on his desktop, but he could not get past the login page, as it had not been configured to his account. A Helpdesk request to IT resulted in him being able to sign in and contact colleagues. However, when he tried to call members of the Petroc team supporting him, he was confronted with a generic pop-up error message to “contact operational support for further information.”

After unsuccessfully calling ICT support, he stopped the tests. The operational support manager was tasked with finding someone in ICT to help:

[W]e’ve had to sort of drive and chase every, sort of, step…. At the moment the IT is the stumbling block… Our managers are running around with their own priorities and I don’t know who will practically help us… You just don’t feel – it hasn’t been allocated to anyone… Our IT requests just go to a generic call desk.

With no response to email requests and continuing demand on the clinic team, progress on developing the virtual consultation service stalled. After two months of chasing, a member of the ICT team investigated the issue. It emerged that, while Skype for Business was available as part of the NHS Mail integration, the default configuration was for internal communications. External communication required additional enterprise licenses, of which the trust had a limited number.

After some weeks, the application on SB’s clinic laptop was successfully configured to enable external video calls. SB managed to run some dummy calls, followed by a single video consultation with a carefully selected patient, which went well. Based on his experience over the last 18 months, he remained concerned about the reliability of the technology and routine use within this busy clinic.

Similar challenges confronted other services at Southern Health. One clinician resorted to purchasing an iPad and using Facetime (with patients who owned Apple devices), thus bypassing the NHS technical infrastructure. All teams expressed a pessimistic outlook on the prospect of routinely running video appointments. While there was no active resistance to using the technology, there was perceived to be an absence of senior-level engagement and leadership:

The technology is there. It is ready and waiting. But we need someone to drive it as their own priority getting these clinics up and running and looking at the various technical issues that come with it… and ironing those out.

Physiotherapist

The subcase illustrates a key finding: that the contingent and emergent qualities of infrastructure mean that it will never be possible to develop a standard pathway to implementation. While support from Petroc Health played an important role, this could not make virtual consultations happen locally. Indeed, this example illustrated that spreading the model to other sites needs to center on the process of translation in the new site, which will never be fully scripted by generic models and implementation guides.

## Discussion

### Main Findings

This paper has described four detailed subcases within a wider case study of scaling up video consultation services in a multi-site London hospital trust and spreading this model to other UK sites. The subcases illustrate how “boring things” (such as internal procedures, locally-endorsed standards, aspects of software functionality, mundane administrative issues such as room bookings, general pressures on the system, cash-constrained departments fighting their corner, and historical decisions to invest in particular platforms) influenced the fortunes of a technological innovation. Infrastructural challenges included intricacy and lack of dependability of the installed base; interdependencies of technologies, processes, and routines, such that a fix for one problem generated problems elsewhere in the system; the inertia of established routines; the constraining effect of budgets and legacy systems; and delays and conflicts relating to regulatory and professional standards, especially around clinical quality and safety.

Our rich ethnographic data revealed the importance of entrepreneurial clinicians, who were committed to introducing new technology to deliver excellent care and who made persistent and creative efforts to interest, enroll, and mobilize others to help align technologies, standards, routines, and processes in the pursuit of that goal. Despite these efforts, and notwithstanding, prevailing, master-narratives of efficiency, standardization, and control, the health information infrastructure in Petroc Health, in particular, was revealed as heterogeneous and unstable, with the various elements pulling apart (drift) and an absence of effective central control (distribution), resulting in what Berg [47] has called:

A fragile, never static equilibrium, characterized by never ending frictions, loose ends, and unforeseen consequences.

Our findings affirmed previous research, which showed that when video consultations work, they can be clinically high-quality and safe. However, for this eventuality to be achieved, the physical (material) and knowledge-practice (nonmaterial) conditions of possibility must be adequate. When (and to the extent that) these infrastructural preconditions are inadequate (eg, when technical breakdown precludes or compromises a video consultation), the purposive interactions of clinical care become awkward and risky. The clinician’s lack of trust in the reliability of the infrastructure is accompanied by a constant undercurrent of anxiety about being unable to care for patients to appropriate standards. As one reviewer commented, “we seem to have created a new form of professional shame.”

To the extent that the precarious infrastructure that characterized our study sites was able to support the innovation and maintain excellence in clinical care, this was in no small part due to creative and persistent work undertaken by individual human actors to bridge, through human and technical workarounds, the gap between standardization and contingency. This articulation work was never-ending; it often had a political component; and it sometimes succeeded only to the extent that the innovation remained confined to a local setting, protected from the wider system. In some cases, the gap between standardization and contingency could never be bridged (eg, when administrative permissions to use a technology were not forthcoming or when internet connectivity proved rate-limiting), resulting in suspension or abandonment of the project.

### Strengths and Limitations

This study has both empirical and theoretical strengths. Empirically, it is based on extensive ethnographic fieldwork conducted over many years and oriented to explore the contingent and emergent qualities of health care infrastructure. The study’s theoretical strength is the extension of Star’s foundational work on infrastructure to include a central emphasis on its institutional elements. We have combined different but related theoretical perspectives, namely, strong structuration theory, neo-institutional theory, social practice theory, and aspects of actor-network theory, and applied these to a rich, longitudinal dataset to tease out how the institutional aspects of information infrastructures emerge and unfold over time.

The limitations of this study include the question of how far we can generalize from a single, unique case to make claims about infrastructure more generally. The five defining components of health information infrastructure set out in the introduction were built on, refined, and developed to explain our empirical data; they may not explain all aspects of such infrastructure in all contexts. We encourage others to apply and extend our theoretical framework to contrasting countries and settings.

### Comparison With Previous Literature

Our work resonates strongly with Star’s original theoretical work on information infrastructure [1,2], as well as that of other founding scholars who have taken a social practice perspective on technology [6,17,24,47].

Hartswood et al, drawing on various ethnographic studies of health information systems in the United Kingdom, introduced the notion of “design in use,” in which design is reconceptualized from a discrete phase that occurs before the technology is implemented to a never-ending effort to achieve and maintain embedding by coadapting the technology and the work processes it aims to support. Facilitated partnerships between ICT specialists and clinician-users are needed to address [48]:

…the ongoing struggle of making this particular system work for these particular users, in this particular workplace and at this particular time.

These authors emphasize the importance of making ICT work more visible to nontechnical users, allowing them to take a more informed leadership role, and, conversely, how making clinical work visible to ICT specialists allows the latter to become attuned to the nuances of technology use, thereby understanding the importance (or not) of particular features or adaptations.

Grisot et al describe a 10-year ethnography of the design, development, and initial use of a web-based solution for patient-hospital communication at a Norwegian hospital [6]. Like the solutions in our case studies, this began as a bottom-up initiative from a small and motivated team, though in the Norwegian case, the entrepreneurs were ICT, not clinical, staff. Numerous challenges and setbacks are described, but overall, the solution grew gradually and in a locally adaptive way as the ICT team assessed and addressed specific user needs and use cases. The authors conclude that ICT innovations that are part of the infrastructure cannot merely be “installed.” Instead, they must be organically “cultivated” in a way that acknowledges changing organizational needs and the inertia and constraints of the installed base [6]. Maniatopoulos et al had similar findings when studying the spread of new diagnostic technology for breast cancer across NHS oncology and pathology units. They concluded that [24]:

It is difficult - if not impossible - to propose a standard pathway for its [lymph node biopsy technology’s] embedding into healthcare practice.

These findings from other research groups mirror our own in highlighting the need to create spaces in which generic elements of solutions can be adapted to local needs and circumstances. However, they differ in that, in our case study, there was a tendency for clinical and informatics staff to talk past one another, with the former (at least to some extent) failing to fully appreciate the technical elements of infrastructure and the latter not fully appreciating the clinical (eg, quality and safety) aspects. This highlights the need to positively promote shared interests and mutual comprehension between the different groups essential to processes of implementation. The scaling-up program for video consultations is, however, at an early stage in its evolution, and there is scope for introducing additional measures to support design-in-use and emergent cultivation. However, this would require, at the very least, attention to the severe staffing shortages that underlie long response times in some participating sites.

### The Institutional Elements of Infrastructure

The UK National Health Service was established in 1947 and remains a cherished national institution among both professionals and the public [49]. Using Scott’s taxonomy [28], the institutional pillars which support the NHS are threefold: formal (including legal) regulations, enduring professional values and codes of conduct, and expected patterns of behavior. Our data revealed that all three pillars are retained and consolidated in the technical elements of NHS information infrastructure as well as in the habitus of NHS staff. This was evident in the nonnegotiable way that rules and regulations (regarding privacy and data protection) were imposed, often in a way that could not be materially overridden, in clinicians’ commitment to providing a “professional” service and the lengths to which they were prepared to go to prevent “embarrassing” violations of the standards they set themselves, and in the long-established patterns of action and interaction that delivered traditional outpatient services. It is for these institutional reasons, of legitimacy and support, and not merely for technical connectivity issues, that the information infrastructure of the NHS has proved so hard to change and why the implementation of new infrastructure often plays out in political ways (eg, as battles between clinical departments, ICT departments, and top management).

UK policy on health technology is currently placing considerable emphasis on light IT innovations such as apps and devices, which are presented as having the potential to improve the quality and efficiency of care. For example, the NHS Innovation Accelerator was established in 2015 [50]:

To speed up adoption of innovations which have proven potential for high impact throughout the NHS and wider healthcare economy.

Such an approach, which places limited emphasis on cultivating the underlying infrastructure with which such novel technologies will need to connect, is deterministic and naïve. Critics have, for example, condemned the new “doctor-in-your-pocket” smartphone video consultation service mentioned in the introduction [11] as both unsafe (because the symptom-checker app is alleged to be unreliable) and unethical (because the service is marketed to young adults, allegedly taking this group and leaving the NHS to care for older and sicker individuals) [51,52]. While a full analysis of this particular controversy is beyond the scope of this paper, the question arises as to whether this controversial service has succeeded precisely because it is not merely technology-light but also regulation-light and value-light. In other words, it has been designed to bypass, rather than integrate with, the institutionalized infrastructure of the NHS.

The challenge of spreading technological innovation through a national public health care system raises the question of how to balance (on the one hand) providing a high degree of stability and commonality through typified inbuilt models, categories, and processes, and (on the other hand) providing sufficient leeway for flexibility to accommodate local contexts (including clinical and geographical particularities, historical path dependencies, and local political agendas) and also allowing the clinician to exercise the necessary autonomy during the consultation. Ellingsen and Monterio observe that “extensive local adaption does not scale, resulting in constraints stemming from one local setting spilling over to the next” [53]. Either we must abandon completely the vision of scaling up innovations that have worked in one setting, or we need to rephrase the scale-up challenge in a more nuanced way, for example: how can we build leeway into technologies to maximize scope for adaptation to different local infrastructures and (at the point of technology use) clinical microcontexts?

### Conclusion

This paper has revisited Star’s classic work on information infrastructure, has developed its theoretical underpinnings particularly concerning institutional influences and has used a contemporary case study to illustrate its continued relevance to the challenges of medical and health informatics. In a 2001 review paper, Orlikowski and Iacono showed that most information system research published at the time had treated technologies in isolation (eg, as tools), while only a small fraction had studied them as ensembles, that is, as part of wider socio-technical systems [54]. We have recently produced similar findings in a health care context (Papoutsi et al, paper submitted). The empirical findings presented in this paper emphasize the importance of an ensemble view of technologies and show how, particularly in a public-sector health care setting, that ensemble incorporates and reproduces institutional rules, norms, and ways of working.

These findings suggest that much could be learned from further ethnographic studies of health information infrastructure in different contexts. While the popular randomized trial design has its place, it tends to reinforce a tool rather than ensemble view of technology and downplay the importance of infrastructure. A case study design is needed to illustrate, for example, how newly introduced technology is initially equivocal [55]. However, through much local negotiation, interpretation, and on-the-job support can become both technologically and institutionally stabilized [56].

Our findings also suggest that innovators and change agents who wish to introduce new technologies in health services and systems could distill some working principles from our retheorization of infrastructure. First, it is essential to attend to materiality, such as to expect bugs and breakdowns and prioritize basic dependability over advanced functionality. Second, the technological artifact should be considered relationally and as part of a system, rather than as an isolated tool or function. Third, it should be remembered that technology-supported work is cooperative and embedded in organizational routines, and that clinical routines are embedded, sometimes inextricably, in other clinical and administrative routines. Fourth, innovation should generally occur incrementally and organically, with careful attention to technological and socio-cultural legacies. Finally, the institutional nature of infrastructure suggests that it is important to attend not just to standards but to where these standards come from, whose priorities and interests they represent, and whether there is sufficient leeway for them to be appropriately adapted to different local conditions.

## Abbreviations

CPU: central processing unit
ICT: information and communication technologies
IT: information technology
NHS: National Health Service
TST: Transforming Services Together
UAT: User Acceptance Testing
VDI: virtual desktop infrastructure
VOCAL: Virtual Online Consultations—Advantages and Limitations
